# Nonuremic calciphylaxis associated with FGFR inhibitor futibatinib: A novel therapeutic role for epoprostenol

**DOI:** 10.1016/j.jdcr.2026.09.014

**Published:** 2026-09-15

**Authors:** Alexa Kassels, Kimiya Ghassemzadeh, Sophia Ederaine, Luke Horton, Pashtoon Murtaza Kasi, Janellen Smith, Michelle S. Min

**Affiliations:** a Department of Dermatology, University of California, Irvine, Irvine, California; b Department of Oncology and Therapeutics Research, City of Hope, Irvine, California

**Keywords:** calciphylaxis, drug reaction, epoprostenol, FGFR inhibitor, futibatinib

## Introduction

Calciphylaxis is a rare, life-threatening condition characterized by painful necrotic skin lesions resulting from small-vessel calcification, intimal fibrosis, and thrombosis.^1^^,^^2^ It most commonly affects patients with end-stage kidney disease. However, non-uremic calciphylaxis can occur in the absence of end-stage kidney disease due to other sources of hyperphosphatemia and dysregulated calcium-phosphate metabolism.^1^^,^^2^ Several recent case reports suggest a link between calciphylaxis and fibroblast growth factor receptor (FGFR) inhibitor therapy, with warnings now added to drug package inserts.^2^, ^3^, ^4^, ^5^, ^6^ To our knowledge, there is only one case report regarding calciphylaxis secondary to futibatinib, a novel FGFR inhibitor approved for treating cholangiocarcinoma.^6^ In this report, we describe a case of refractory calciphylaxis in a patient receiving futibatinib who demonstrated a significant response to epoprostenol therapy.

## Case report

A woman in her 60s with stage IIIc intrahepatic cholangiocarcinoma was urgently referred from an outside hospital for evaluation of potential vasculitis of the lower legs. Two weeks prior to presenting to dermatology, futibatinib had been initiated. The patient complained of severe pain associated with worsening purpuric plaques of the lower legs. Physical examination revealed black necrotic, stellate plaques with surrounding livedoid violaceous erythema of bilateral lower legs (Fig 1, *A*). Calcium and phosphorous levels had recently peaked at 11.1 mg/dL (reference range 8.6-10.0 mg/dL) and 7.4 mg/dL (2.5-4.6 mg/dL), respectively, after the patient had received calcium supplementation for tumor lysis syndrome days prior. Creatinine, blood urea nitrogen, and parathyroid hormone values were within normal limits.

**Fig 1:**
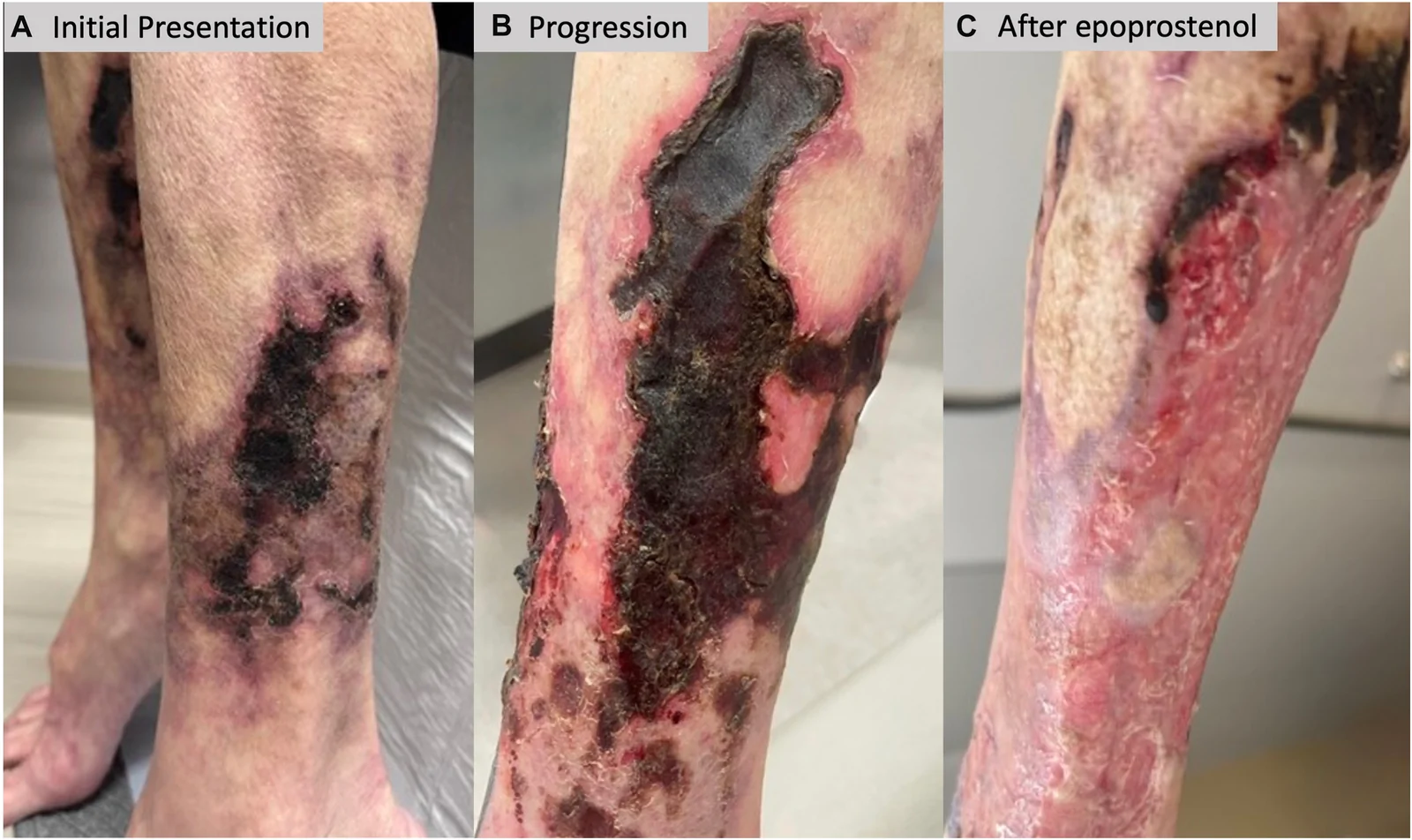
Clinical Images. **A,** Initial black necrotic plaques with surrounding livedoid violaceous erythema and some areas of retiform purpura on the bilateral lower legs. **B,** Increasingly inflamed borders of ulcerated plaques. **C,** Substantial healing of ulcers of lower legs following epoprostenol infusion.

Given severity of pain, skin biopsy was initially deferred. X-ray showed faint vascular calcifications present in both calves, consistent with calciphylaxis (Fig 2, *A*). The patient was immediately started on IV sodium thiosulfate (a calcium chelator) 25 mg three times weekly and sevelamer (an oral phosphate binder) 800 mg three times daily. When calciphylaxis continued to progress, oncology agreed to hold futibatinib.

**Fig 2:**
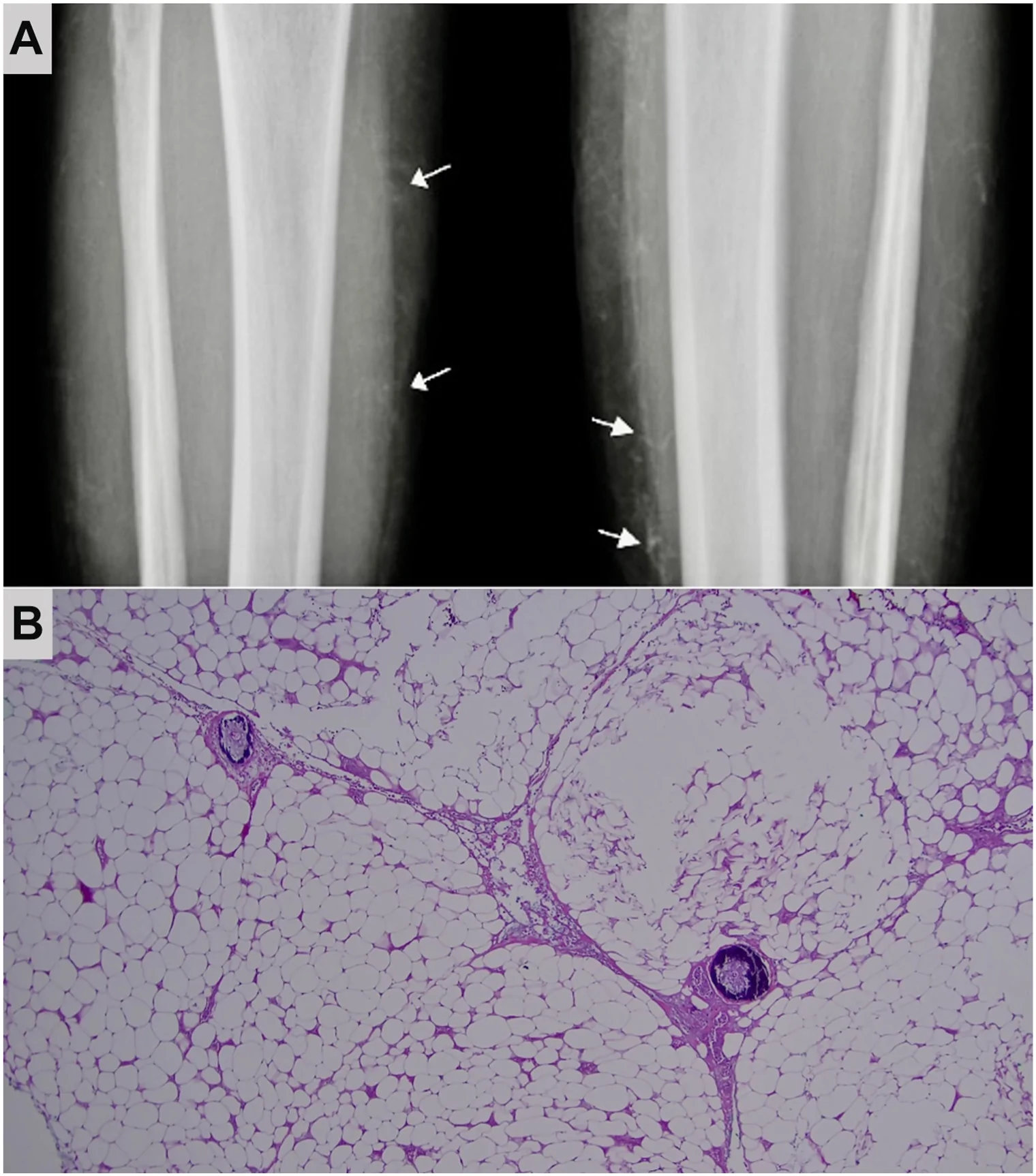
X-ray and histopathology image. **A,** X-ray showing faint vascular calcifications (*arrows*) present in both calves consistent with calciphylaxis. **B,** Skin biopsy from the thigh revealed lobular fat necrosis with calcification of vessels consistent with calciphylaxis.

One month later, ulcer borders appeared increasingly inflamed (Fig 1, *B*). Aspirin, pentoxifylline, sildenafil, and a short course of prednisone (max dose 30 mg daily) were initiated. Due to worsening pain and ulcerations, the patient was admitted 5 weeks later for xenograft placement, wound care, and pain management. Unfortunately, she developed persistent, symptomatic hypocalcemia and hypophosphatemia during hospitalization, necessitating discontinuation of sodium thiosulfate and sevelamer.

Two weeks later, fixed livedoid racemosa extended to bilateral thighs, previously uninvolved, with new calcified subcutaneous nodules (Fig 3, *A*). Skin punch biopsy showed lobular fat necrosis with calcification of small vessels in the reticular dermis, consistent with calciphylaxis (Fig 2, *B*). Given progression, the patient was admitted for a 72-hour continuous epoprostenol infusion (0.005 mg/mL), initiated at 2 ng/kg/min and slowly titrated up to 8 ng/kg/min. Within hours of epoprostenol monotherapy, ulceration progression on bilateral lower legs halted and livedoid changes receded from the thighs with marked improvement photographed just 3 days later (Figs 1, *C* and 3, *B*).

**Fig 3:**
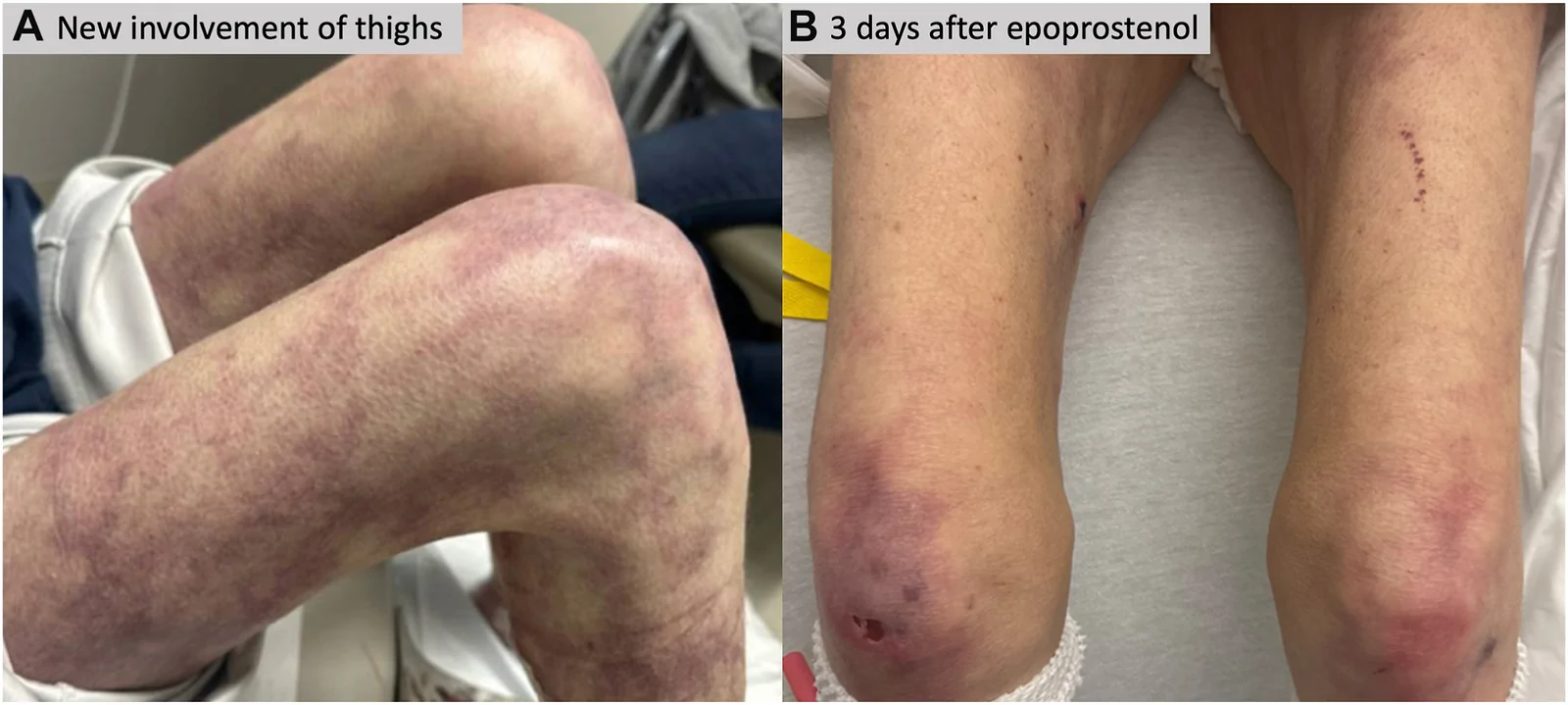
Clinical Images. **A,** Fixed livedoid racemosa extended to the thighs with new development of calcified subcutaneous nodules despite prior standard of care therapy. **B,** Livedoid changes rapidly receded from the thighs within 3 days of epoprostenol treatment.

The patient demonstrated continued healing. Unfortunately, she suddenly passed weeks later from unrelated complications.

## Discussion

This case highlights a presentation of nonuremic calciphylaxis induced by futibatinib, an FGFR inhibitor. Calciphylaxis is caused by calcification and occlusion of blood vessels in the dermis and subcutaneous adipose tissue, leading to tissue ischemia and necrosis. Notably, hyperphosphatemia, a significant risk factor for calciphylaxis, occurs in 77% of patients receiving FGFR inhibitors.^6^ While the mechanism linking FGFR inhibitors to calciphylaxis remains unclear, it has been proposed that hyperphosphatemia alters calcium-phosphate product, leading to vascular calcification. FGFR inhibitors may also induce compensatory elevations in FGF 23, a phosphate-regulating hormone implicated in vascular calcification.^2^ Of note, our patient also received calcium supplementation for tumor lysis syndrome days after initiating futibatinib, which may have further increased susceptibility to calciphylaxis.

The presence of black, necrotic stellate plaques with surrounding livedoid changes should raise a high index of suspicion for calciphylaxis in patients on FGFR inhibitors. In reported cases of FGFR inhibitor-associated calciphylaxis (including agents such as pemigatinib, erdafitinib, and futibatinib), lesions have predominantly involved the lower extremities, particularly the thighs and lower legs.^2^, ^3^, ^4^, ^5^, ^6^ In comparison, classic calciphylaxis commonly favors areas of increased adiposity, particularly the lower abdomen, buttocks, and inner thighs, although distal extremity involvement can be observed in advanced disease.^7^^,^^8^

Calciphylaxis is associated with high mortality, making early recognition and prompt treatment essential to improve outcomes. Across reported FGFR inhibitor-associated cases, management has generally included immediate discontinuation of therapy, aggressive wound care, and optimization of calcium-phosphate balance via adjunctive therapies (eg, sodium thiosulfate, sevelamer).^4^, ^5^, ^6^ Hyperbaric oxygen therapy and perfusion-enhancing agents (eg, pentoxifylline, sildenafil) are also used in select cases to promote healing and limit ischemic injury.

We report novel use of epoprostenol as an adjuvant treatment for those with refractory calciphylaxis or when conventional therapies cannot safely be administered. Given disease progression on standard therapies with subsequent discontinuation, and rapid improvement within hours of epoprostenol monotherapy, we suspect epoprostenol played a major role in our patient’s improvement. Epoprostenol is a prostacyclin analog approved for pulmonary arterial hypertension and used off label as rescue therapy for severe Raynaud’s crises. It increases tissue perfusion, thereby limiting ischemic injury. Administered as a continuous 72-hour infusion with close monitoring in the intensive care unit setting, multidisciplinary coordination with pulmonology is advised. Contraindications to epoprostenol include severe left ventricular systolic dysfunction, pulmonary edema, and hypersensitivity to prostacyclin analogs. Adverse reactions include headache, hypotension, cardiac complications, and flu-like symptoms.^9^

To our knowledge, there are no other reported cases of epoprostenol used to treat calciphylaxis, though iloprost, another prostacyclin analog, has been described in conjunction with sodium thiosulfate.^10^ The adjunct use of epoprostenol appears biologically plausible for refractory disease, given its vasodilatory effects.

Overall, this case highlights the association between FGFR inhibitors such as futibatinib and calciphylaxis. In these cases, calciphylaxis may present unconventionally: kidney disease can be absent, lower legs tend to be involved, and typical therapies might not be suitable. When clinical suspicion for calciphylaxis is high, we recommend immediate cessation of FGFR inhibitor therapy when feasible. Finally, we propose epoprostenol as an effective therapeutic option for calciphylaxis as an adjuvant or for refractory disease to promote vasodilation and oxygenation of ischemic tissue.

## Conflicts of interest

Dr Min is on the advisory boards of Horizon and BMS. She is an investigator for Amgen, AZ, BI, BMS, and Priovant. Drs Kassels, Ederaine, Horton, Kasi, Smith, and Author Ghassemzadeh have no conflicts of interest to declare.
